# Combined vitrectomy, near-confluent panretinal endolaser, bevacizumab and cyclophotocoagulation for neovascular glaucoma — a retrospective interventional case series

**DOI:** 10.12688/f1000research.26879.2

**Published:** 2021-03-02

**Authors:** Piotr Strzalkowski, Alicja Strzalkowska, Winfried Göbel, Nils A. Loewen, Jost Hillenkamp

**Affiliations:** 1 Department of Ophthalmology, School of Medicine, University Hospital Wuerzburg, 97080, Germany

**Keywords:** Neovascular glaucoma, integrative surgical approach, iris neovascularization

## Abstract

**Background:** Neovascular glaucoma (NVG) is a severe, potentially blinding disease and a therapeutic challenge. The purpose of this study was to evaluate the safety and efficacy of an integrative surgical approach to neovascular glaucoma.

**Methods: **Retrospective analysis of a one-year follow-up of a consecutive interventional case series of NVG. Eyes underwent transscleral cyclophotocoagulation, pars plana vitrectomy, near-confluent panretinal photocoagulation, and intravitreal bevacizumab. Phakic eyes underwent concomitant cataract surgery. Best-corrected visual acuity (BCVA, logMAR), intraocular pressure (IOP, mmHg), number of glaucoma medication, visual analog pain scale (VAPS, 0-10) were recorded at baseline, and 1, 3, 6, and 12 months. Blind eyes were excluded.

**Results:** Seventy-seven eyes of 77 patients (45 male, 32 female, mean age 73.6±12.2 years) were included. NVG underlying conditions included retinal vein occlusion (41.6%), proliferative diabetic retinopathy (35.1%), central retinal artery occlusion (19.5%), and ocular ischemic syndrome (3.9%). Mean IOP decreased postoperatively from 46.3±10.1 mmHg to 14.5±7.9 mmHg (p<0.001), glaucoma medication from 4.7±1.3 to 1.8±1.8 (p<0.001), and VAPS from 6.0±1.8 to 0. BCVA remained unchanged. Postoperative intraocular inflammation had resolved in all eyes at the one-month follow-up. 71.4% (55/77) eyes did not require additional major interventions during follow-up.

**Conclusions:** A single, comprehensive surgery session lowered IOP significantly, reduced GMS, and controlled pain.

## Introduction

Neovascular glaucoma (NVG) is a serious complication of a variety of ocular and systemic conditions. Neovascularization is the formation of abnormal blood vessels in an abnormal location triggered by an imbalance of anti-angiogenic and proangiogenic factors caused by retinal ischemia ^1^. NVG accounts for 3.9 to 9.2% of all new glaucoma diagnoses ^2– 4^. According to the Federal Statistical Office of Germany, NVG’s age-specific incidence in Germany in the age group from 45 to 64 years is 8 per 100,000. It increases to 24 per 100,000 in subjects older than 64 years ^5^. The incidence of NVG varies depending on the etiology of retinal ischemia. For central retinal vein occlusion (CRVO) the reported incidence is 16% ^6^, for proliferative diabetic retinopathy (PDR) 21.3% ^7^, for central retinal artery occlusion (CRAO) 14.5% ^8^, and ocular ischemic syndrome (OIS) 12.9% ^9^, respectively. Carotid artery obstructive disease and fistulas are additional, extraocular vascular causes of retinal ischemia ^10^. Early recognition and treatment of NVG are imperative to prevent aggressive evolution with severe vision loss and intractable pain that can require enucleation within a few months ^11^. NVG also carries a poor prognosis for general health: remarkably, the expected lifespan of patients with NVG decreased by 52% compared to an age-correlated normal population, which corresponds to 6.5 years. In diabetics with NVG, the expected lifespan was reduced even more significantly by 72% (5.1 years in this subgroup) ^12^.

In 1994, Miller *et al.* showed that retinal laser coagulation of primates could lead to CRVO and subsequent retinal ischemia with iris neovascularization ^13^. In this model, neovascularization was mediated by vascular endothelial growth factor (VEGF) and correlated to its concentration. Several members of the VEGF family have since been identified, including VEGFA, VEGFB, VEGFC, VEGFD, and the placental growth factor (PLGF) with specific receptor-binding patterns. VEGFA primarily binds to VEGFR2, the activation of which stimulates neovascularization, relevant to NVG, and angiogenesis, the formation of normal blood vessels in normal development. In the eye, VEGF-A is produced by the retinal pigment epithelium, retinal ganglion cells, astrocytes, the endothelium, photoreceptors, and Müller cells ^14– 17^. Retinal hypoxia upregulates VEGF primarily via the hypoxia-inducible factor (HIF-1α and HIF-2α). The concentration of HIF increases when hydroxylases are inhibited during hypoxia so that HIF is not degraded by the proteasome ^18^. HIF binds to the hypoxia-responsive element (HRE) of the VEGF gene in the nucleus leading to its upregulation ^19^.

Although the mechanism by which NVG emerges is hence relatively well understood, there is no consensus on how to best initiate treatment to address the different aspects. Treatment steps include lowering of IOP (topical and systemic glaucoma medications, glaucoma drainage implants, cyclodestruction) ^20– 24^, anti-inflammatory treatment (topical or intraocular steroids), reduction of retinal ischemia (panretinal photocoagulation (PRP)) ^25, 26^, and inhibition of VEGF ^27– 29^. Vitrectomy with PRP and silicone oil tamponade may also reduce IOP in eyes with NVG ^30, 31^.

Here, we evaluated the safety and efficacy of an integrative combined surgical approach for evolving NVG that combined pars plana vitrectomy, near-confluent full-scatter panretinal photocoagulation, off-label use of intravitreal bevacizumab, and transscleral cyclophotocoagulation in a single surgical session. We hypothesized that such a combined surgical approach for NVG would reduce IOP, medication, pain, reduce the number of necessary outpatient visits, and prevent the necessity of further surgeries.

## Methods

### Ethical statement

Our retrospective study was reviewed and approved by the Ethics Committee of the University of Würzburg (reference: 9/17-sc, dated February 3, 2017) and a clearance certificate for retrospective data evaluation was issued (reference: 20180108 02, dated January 30, 2018). For retrospective anonymized data analysis, patient’s consent was not necessary. The Declaration of Helsinki, the Good Clinical Practice (GCP) guidelines and applicable data protection regulations were complied with.

### Study design

We included all patients between October 2014 and August 2019 with NVG who met the inclusion criteria in this consecutive interventional case series.

Inclusion criteria were: 1) neovascularization of iris (NVI) or neovascularization of the angle (NVA), 2) IOP > 21 mmHg, 3) best-corrected visual acuity (BCVA, logMAR) ≧ light perception, and 4) 18 years or older.

All patients were treated at the University Eye Hospital in Würzburg, Germany. The combined intervention was part of routine patient care at our clinic and all patients with decompensated neovascular glaucoma and preserved visual function received this intervention. BCVA, intraocular pressure (IOP, mmHg), the number of glaucoma medication, visual analog pain scale (VAPS, 0-10) was recorded at baseline and at follow-up visits at one, three, six, and 12 months as part of routine care. Iris and anterior chamber neovascularization were graded using the Weiss and Gold rubeosis grading system ^32^. Hypotony was defined as IOP ≤ 5 mmHg with hypotonous maculopathy, choroidal folds, or optic neuropathy ^33^. Phthisis bulbi ^34^ was defined as IOP ≤ 5 mmHg in a shrunken eye with worse than hand motion vision with or without pain containing atrophic and disorganized intraocular structures. Success was defined as lOP ≤ 21 mmHg or IOP reduction ≥ 30% from baseline, with or without glaucoma medication and without vision loss ^35^.

All patients underwent decimal visual acuity testing, which was converted to a logMAR scale. Counting fingers (CF), hand movements (HM), light perception (LP), and no light perception (NLP) were converted into logMAR units 1.9, 2.3, 2.7, and 3.0, respectively ^36^.

We retrospectively analyzed all patients treated with neovascular glaucoma at our clinic via the electronic hospital information system SAP®. The medical records of the treated patients were analyzed individually. The initial diagnosis of neovascular glaucoma and hospitalization served as the starting point. The cardiovascular risks were taken from the anesthesia premedication form.

### Patient treatment and follow-up

***Surgical technique.*** All eyes underwent transscleral cyclophotocoagulation (810 nm diode laser, 360 degrees treatment to pop threshold with 20 spots and leaving out 3 and 9 o’clock), standard 3-port 23 gauge pars plana vitrectomy with a detachment of the posterior vitreous if not already present, near-confluent full-scatter panretinal photocoagulation (PRP) applied under indentation in all four quadrants from the vascular arcades to the ora serrata, intravitreal application of 0,1 mL of bevacizumab (Avastin®️ 25 mg/1 mL, Roche Pharma, Switzerland), and air tamponade. Phakic eyes underwent concomitant cataract surgery. The operation was carried out either with a retrobulbar block or general anesthesia.

***Retreatment.*** At follow-up, all eyes with elevated IOP were treated following an escalation scheme. First, glaucoma medications were increased to what was maximally tolerated. Eyes were then treated with transscleral cyclophotocoagulation (810 nm diode laser, 360 degrees treatment to pop threshold with 20 spots). Eyes that failed to respond to cyclophotocoagulation with a significant IOP reduction and had retained ambulatory visual acuity underwent tube shunt surgery. Repeated vitrectomy, including fill-in PRP, transscleral cyclophotocoagulation, and off-label use of intravitreal bevacizumab was applied to eyes with elevated IOP and dense vitreous hemorrhage. Fill-in PRP was applied when PRP could not be completed during surgery in eyes with extensive intraretinal hemorrhage. Further intravitreal injections of VEGF inhibitors were only applied for clinically significant non-ischemic macular edema and BCVA ≥1.1 logMAR.

***Patients lost to follow-up.*** All patients who failed to attend follow-up visits completed a standardized telephone survey that contained the following questions: What was the main reason for not following up? What is your current vision function? How many glaucoma drops are you using? Do you have any eye pain? Are you overall satisfied with the outcome of the treatment?

### Statistical methods

Data analysis was performed using Statistica 13.1 (Tulsa, Oklahoma, United States). The frequency of observations described categorical variables. Continuous variables were described as mean with standard deviation (SD) or median with range (minimum-maximum). Friedman test and Wilcoxon signed-rank test were used to compare data measured on an ordinal scale and continuous variables with non-normal distribution. Evaluation of data normality was performed using the Shapiro-Wilk test. Welch’s t-test for unequal variances was used for IOP between pain versus no-pain group comparison. Categorical variables of the relationship between neovascularization stage and type of intervention were compared using the χ2 test with odds ratio (OR) measurement. Kaplan–Meier curve and log-rank test were used for success analysis. P-values <0.05 were considered significant.

An earlier version of this article can be found on medRxiv (doi: https://doi.org/10.1101/2020.01.19.20017889)

## Results

**Figure 1.**
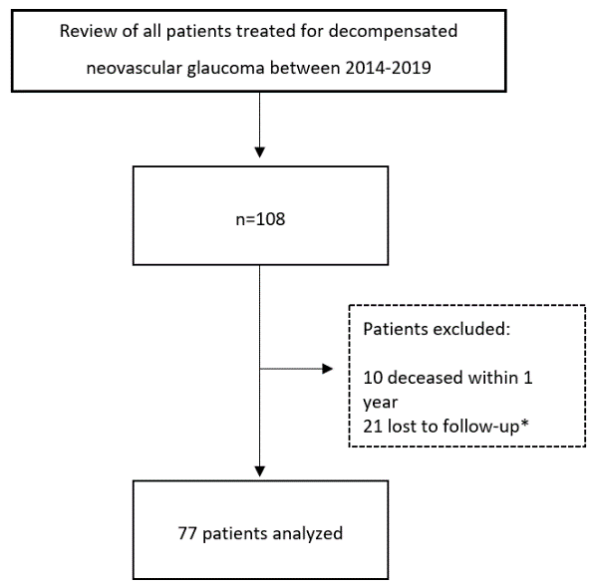
Flow chart. Eyes with no light perception and patients with a history of glaucoma other than neovascular glaucoma were excluded. *Telephone survey with all patients lost to follow-up.

All 77 patients (45 male, 32 female, mean 73.6±12.2 years, range 29–91 years) completed a one-year follow-up and were included in the retrospective analysis ( Figure 1). Conditions that lead to NVG included CRVO 41.6% (32/77), PDR 35.1% (27/77), CRAO 19.5% (15/77), and ocular ischemic syndrome 3.9% (3/77) ^37^ ( Table 1).

**Table 1.** Demographic characteristics of neovascular glaucoma patients.

**Variables**	n = 77
**Age (years)**	73.6±12.2 (range 29–91)
**Gender**	n (%)
Female	32 (41.6)
Male	45 (58.4)
**Laterality**	n (%)
Right	34 (44.2)
Left	43 (55.8)
**Diagnosis**	n (%)
CRAO	15 (19.5)
CRVO	32 (41.6)
PDR	27 (35.1)
OIS	3 (3.9)
**BMI (kg/m ^2^)**	n (%)
<18.5	1 (1.3)
18.5-24.9	19 (24.7)
25-29.9	31 (40.3)
30-34.9	19 (24.7)
>35	7 (9.1)
**NVI stage**	n (%)
Stage 1	0 (0)
Stage 2	9 (11.7)
Stage 3	26 (33.8)
Stage 4	42 (54.5)

BMI, body mass index; CRAO, central retinal artery occlusion; CRVO, central retinal vein occlusion; PDR, proliferative diabetic retinopathy; OIS, ocular ischemic syndrome.

The most common cardiovascular risk factor was hypertension 87.0% (67/77), followed by hyperlipidemia 67.5% (52/77), diabetes mellitus 59.7% (46/77), and smoking 11.7% (9/77). While one patient did not have any cardiovascular risk factors, 24.7% (19/77) had at least one risk factor, and 74.0% (57/77) had multiple risk factors.

The mean body mass index (BMI kg/m²) for the study group was 28.7±5.0 (underweight ≤ 18.5, normal weight = 18.5–24.9, overweight = 25–29.9, obesity ≥ 30 ^38^). 1.3% (1/77) were underweight, 24.7% (19/77) had a normal weight, 40.3% were overweight (31/77) and 33,8% (26/77) obese.

**Figure 2.**
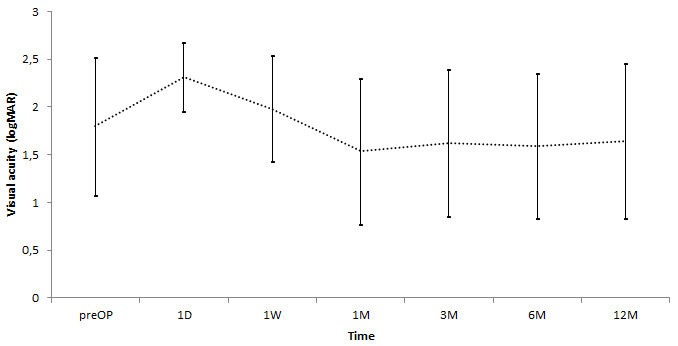
Visual acuity during follow-up (mean ± SD) (p=0.47).

Mean logMAR BCVA was 1.9±0.7 at baseline,1.7±0.8 at one month, 1.8±0.8 at three months, 1.8±0.8 at six months, and 1.8±0.8 at 12 months (p=0.47, Figure 2). At baseline, 35.1% (27/77) of patients and at 12 months, 45.5% (35/77) of patients had an ambulatory visual acuity (≥ logMAR 1.7), respectively, but was not statistically significant (p=0.0931). One eye worsened from LP to NLP at one week, three eyes at six months, and three eyes at 12 months.

**Figure 3.**
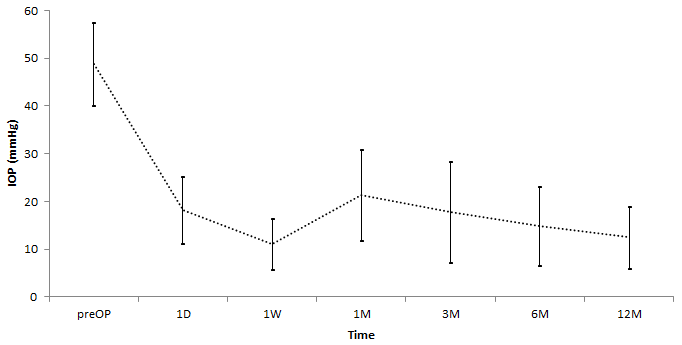
Intraocular pressure (IOP) during follow-up (mean ± SD) (p<0.001).

**Figure 4.**
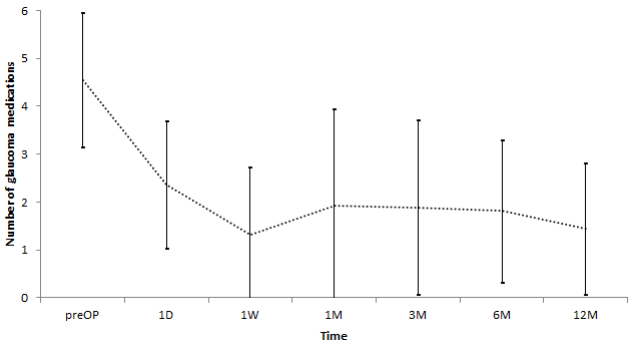
Number of glaucoma medication during follow-up (mean ± SD) (p<0.001).

IOP decreased significantly from 46.3±10.1 mmHg at baseline to 21.4±10.9 mmHg at one month, 18.6±10,7 mmHg at three months, 15.1±7.6 mmHg at six months, and 14.5±7.9 mmHg at 12 months (p<0.001; Figure 3). At one-year follow-up, 89.6% (n=69) of patients had an IOP ≤ 21 mmHg. IOP ≤ 5 mmHg was found in 7.8% (n=6) and tolerated without complications. 96.1% (n=74) presented at baseline with IOP≥30 mmHg. The number of glaucoma medication decreased from 4.7±1.3 at baseline to 1.9±1.9 at one month, 1.8±1.7 at three months, 1.8±1.5 at six months, and 1.8±1.8 (p<0.001; Figure 4) at 12 months.

While 32.5% (n=29) of patients complained of ocular pain at baseline (VAPS: 6.0±1.8), all patients were without pain at all follow-up visits. Patients with pain had a significantly higher baseline IOP of 49.9±9.0 mmHg than patients without pain 44.1±10.3 mmHg (p<0.01).

Early postoperative complications (day 1 to four weeks) included intraocular fibrin 67.5% (52/77), hyphema 20.8% (16/77), choroidal detachment 16.9 (13/77), and corneal erosion 14.3% (11/77). Late postoperative complications (> 1 months) included retinal detachment in 3.9% (3/77) and consecutively a painless phthisis in these 3 cases ( Table 2).

**Table 2.** Early postoperative inflammation and postoperative complications.

Complications	n (%)
Early inflammation/complications	
Extensive fibrin	52 (67.5)
Hyphema	16 (20.8)
Choroidal swelling	13 (16.9)
Corneal erosion	11 (14.3)
Late complications	n (%)
Retinal detachment and Phthisis bulbi	3 (3.9)

At the one-year follow-up, surgical intervention was required in 16.9% (22/77) eyes. Of these eyes, 63.6% (14/22) received vitrectomy, 22.7% (5/22) a Baerveldt glaucoma drainage implant, 4.5% (1/22) a trabeculectomy with mitomycin C and 4.5% (1/22) a keratoplasty. One painful and blind eye needed enucleation.

Repeat transscleral cyclophotocoagulation was performed in 11.6% (15/77). 3.9% (5/77) of patients with a mean BCVA 1.1±0.3 logMAR received an additional 4.0±0.8 anti-VEGF injections.

**Figure 5.**
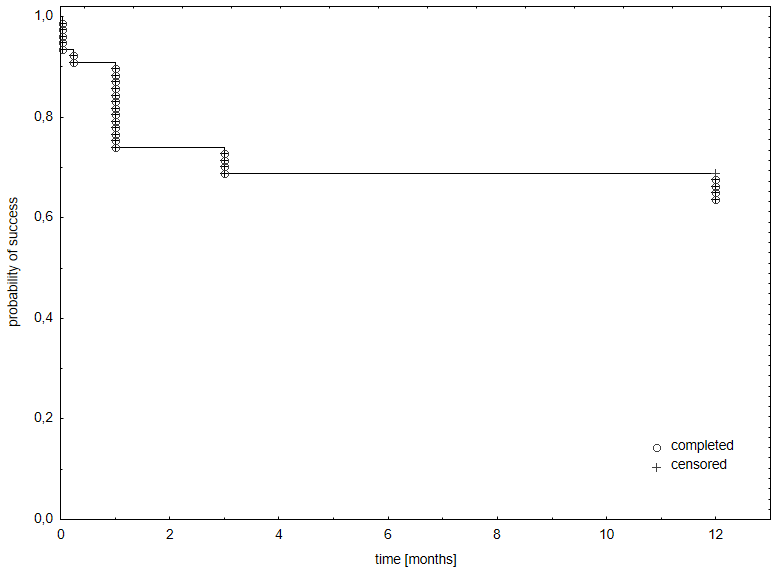
Kaplan-Meier univariate estimates of the probability of success: intraocular pressure (lOP) ≤ 21 mmHg or IOP reduction ≥ 30% from baseline, with or without glaucoma medication, without vision loss.

At baseline, 11.7% (9/77) had neovascularization of the iris (NVI) stage of 2, 33.8% (26/77) stage 3 and 54.5% (42/77) stage 4. The combined average stage was 3.4±0.7 (range 2–4). Patients with stage 4 needed significantly more major interventions compared with stage 3 (OR 25.0, 95% CI 3.09-201.7, p=0.003) and stage 2 (OR 19.0, 95% CI 1.03-347.3, p=0.047). There was no statistically significant difference between the number of interventions required at stage 2 and stage 3 (OR 0.89, 95% CI 0.03-23.9, p=0.9471). Kaplan-Meier analysis showed a probability of success of 65% at one-year follow-up ( Figure 5).

### Lost to follow-up

21 patients were lost to follow-up. 10 patients died during the one-year follow-up period. All 21 patients lost to follow-up completed a telephone survey. The main reasons for missed visits were general health issues in 52.4% (11/21), long-distance to our clinic 38.1% (8/21), and family-related reasons in 9.5% (2/21). 57.1% (12/21) of patients had retained at least ambulatory vision. 71.4% (15/21) used 1.7±1.2 glaucoma eye drops. 95.2% (21/22) reported overall satisfaction with the treatment. All patients were pain-free.

## Discussion

By 1871, NVG was known as “glaucoma haemorrhagicum et apoplecticum” and feared as a consequence of ischemia that quickly led to enucleation due to “hefty ciliary neuralgia” ^39^. In 1963, using improved equipment, Weiss *et al.* found that emerging neovascularization and fibrovascular membranes of the iris and the angle could be observed well before the onset of advanced NVG ^40^, hinting at a window to initiate treatment. Today, the ability to detect neovascularization early is complemented by improved interventions that address both the underlying pathology and elevated IOP. However, these interventions need to be implemented during the early stages of the disease because NVG continues to have a rapid evolution and a poor prognosis for both the eye and the patient ^12^. One study showed that the expected lifespan of patients with NVG decreased by 52% compared to an age-correlated normal population, corresponding to a loss of 6.5 years. In diabetics with NVG, the lifespan was reduced even more by 72% ^12^. In our study, 74% (57/77) of patients had multiple cardiovascular risk factors and were overweight or obese. During follow-up, ten patients died. The high mortality rate is a reminder that any treatment strategy of NVG should aim at keeping the number of necessary follow-up visits and additional interventions at a minimum. We tried to address this need by combining several interventions in a single surgical session.

Retinal ischemia is the primary cause of NVG ^10, 41^. Accordingly, PRP is the standard of care to reduce posterior pole oxygen demand and angiogenic drive while vitrectomy is performed to increase the partial pressure of vitreous oxygen ^42, 43^. We performed lens extraction, pars plana vitrectomy, and endoscopic PRP because of significant media opacities and because endoscopic laser through the pars plana approach facilitates the delivery of 360° near-confluent peripheral retinal laser treatment out to the ora serrata. In our clinical experience, even relatively small areas of retinal ischemia may cause further NVG progression. Therefore, we took care to apply 360° PRP to near-confluence up to the ora serrata. Such extensive treatment would be challenging or impossible using standard PRP with an indirect ophthalmoscope or at the slit lamp.

In the healthy eye, the vitreous body and the iris-lens diaphragm form a relative diffusion barrier that maintains a higher oxygen partial pressure in the posterior chamber than the vitreous overlying the posterior pole. Concurrently, it reduces the diffusion of angiogenic mediators. Recreating a relative diffusion barrier after vitrectomy ^44^ is beneficial and reduces NVI occurrence ^45^. A relative barrier can be achieved with silicone oil ^46^ that reduces the incidence of NVG ^46, 47^. Following this concept, Bartz-Schmidt *et al.* treated 32 NVG patients with pars plana vitrectomy, retinal, and ciliary body photocoagulation, and silicone oil tamponade as eyes were left aphakic. This approach controlled IOP (defined as an IOP between 8 and 21 mmHg) in 72% of patients for at least one year ^30^. In our study we achieved an IOP control in 77.9% and all eyes were pseudophakic and without silicone oil at the conclusion of the surgery, thereby suggesting that silicone oil as a diffusion barrier may not be necessary ^30^.

Our success rate of 65%, defined as an IOP <22 mmHg, with or without glaucoma medication and without vision loss after a one-year follow-up, is similar to success rates reported for glaucoma drainage devices, which range from 62% to 66.7% ^24, 48– 50^. One study reported a 73% success rate in 38 eyes that received a glaucoma drainage device and had relatively few postoperative complications ^51^. However, this report is the exception as others have found ^24, 48– 50^. ( Table 3).

**Table 3.** Literature overview.

Authors	Year	Design	Intervention	n	IOP preOP (mmHg)	Success (%)	Vision loss (%)	Phthisis bulbi (%)
Mermoud *et al.* ^60^	1993	retrospective	Molteno-Valve	60	42.3 ± 13.2	62	48	18
Every *et al.* ^49^	2006	prospective	Molteno-Valve	145	40.1 ± 13.0	72	32	N/A
Yalvac *et al.* ^8, 48^	2007	retrospective	Ahmed-Valve vs Molteno- Valve	38/27	39.5 ± 4.5 vs 39.3 ± 3.9	63 vs 37	23-33	7.9-14.8
Takihara *et al.* ^56^	2009	retrospective	Trabeculectomy + MMC	101	35.9 ± 11.3	62.6	12.9	5.0
Netland *et al.* ^51^	2010	retrospective	Ahmed-Valve ± NVG	38/38	39.1 ± 11.2 vs 43.8 ± 11.0	89.2 vs 73.1	23,7	13,2
Shen *et al.* ^61^	2011	retrospective	Trabeculectomy versus Ahmed-Valve	20/20	47.7 ± 10.2 vs 47.8 ± 11.3	70 vs 65	15 vs 30	5.0
Xie *et al.* ^50^	2019	retrospective	Ahmed-Valve	66	48.23±8.17	66.7	N/A	N/A
Strzalkowski *et al.* ^37^	2021	retrospective	PPV+EL+Avastin+ CPC	77	46.0 ± 10.3	64.6	3.9	5.7

Trabeculectomy has been one of the most important intraocular pressure lowering operations in glaucoma since the 1960s ^89,90^, so this surgical technique was also used in NVG. Different studies have shown a failure rate of up to 80% ^52, 53^. The use of mitomycin C (MMC) and 5-fluorouracil (5-FU) led to a higher success rate and was 54% after 18 months for MMC used intraoperatively and 55% after 35 months for 5-FU ^54^. A combined trabeculectomy and retinal cryotherapy did not seem to improve the surgical success outcome for NVG in diabetic patients ^55^. However, in this study patients had received panretinal photocoagulation prior to the surgery and in contrast to our study IOP ≤ 21 mmHg was the only success criteria.

Neovascular glaucoma is a major risk factor for failure of trabeculectomy ^56, 57^. Chronic blood-retinal barrier insufficiency, for example in advanced diabetic retinopathy, in which endothelial damage and the subsequent release of serum proteins occurs, plays an important role ^58^. Retinal ischemia also leads to the production of inflammatory mediators ^59^, which can lead to filtering bleb scarring and postoperative failure of the trabeculectomy ^58^.

Our integrative surgical approach avoided tube-specific complications (e.g., tube exposure, retraction, corneal touch, obstruction), ranging from 13% to 26% in NVG over five years ^24, 48– 51^. It delivers both retina and glaucoma treatment in a single surgical session and reduces the patient and health care system burden by simplifying postoperative care and follow-up. Our telephone survey with all patients lost to follow-up revealed that the main reasons for missed visits were other (general) health issues in 52.4% and long distance to the clinic in 38.1% but a high subjective satisfaction rate.

Patients with NVI stage 4 needed significantly more major interventions than stage 3 or stage 2, but there was no difference between stage 2 and stage 3. This finding suggests that early and comprehensive treatment may be more beneficial than a stepwise treatment strategy.

It is worth noting that IOP reduction can also be achieved without cyclodestruction applying only pars plana vitrectomy, lensectomy with a preserved anterior capsule, and panretinal endophotocoagulation as reported by Kinoshita *et al.* ^31^. However, this study included only 13 eyes with a lower mean preoperative IOP of 29 mmHg as in our study. By contrast, other studies that only used anti-VEGF agents as primary treatment ^28, 62^ reported failure rates up to 88% ^62^. Anti-VEGF agents may be best used as an adjuvant treatment ^29^. Consistent with our findings, a study that used pars plana vitrectomy, endoscopic peripheral panretinal photocoagulation, and endocyclophotocoagulation (ECP) also achieved an IOP reduction which was more effective than panretinal photocoagulation, intravitreal bevacizumab, pars plana vitrectomy, and trabeculectomy with mitomycin C or Ahmed valve placement. However, the authors reported a higher phthisis rate of 7.4% ^63^.

Because of the underlying ocular disease, visual function remained low in most eyes in our study.

There are limitations to our study. Because the integrative surgical approach described here was the primary practice pattern, there was no control group. This limited us to an intragroup comparison of before versus after treatment data. As a retrospective study, it can only inform on future prospective studies’ parameters and design and help formulate, but not answer, hypotheses about associations between treatment and outcomes.

In conclusion, this study shows that NVG can be controlled in most cases by an integrative surgical approach delivered in a single surgical session that combines transscleral cyclophotocoagulation, cataract removal, pars plana vitrectomy, near-confluent full-scatter panretinal photocoagulation, and intravitreal bevacizumab. Patients with advanced iris neovascularization required significantly more additional interventions. The described approach lowered IOP significantly, reduced the number of glaucoma medications, and controlled pain.

## Data availability

### Underlying data

Open Science Framework: Combined vitrectomy, near-confluent panretinal endolaser, bevacizumab and cyclophotocoagulation for neovascular glaucoma — a retrospective interventional case series. https://doi.org/10.17605/OSF.IO/QTCGV ^37^.

Data are available under the terms of the Creative Commons Zero “No rights reserved” data waiver (CC0 1.0 Public domain dedication).
