# Supplementary material for: Combined vitrectomy, near-confluent panretinal endolaser, bevacizumab and cyclophotocoagulation for neovascular glaucoma — a retrospective interventional case series
Source: F1000Res. 2021 Mar 2;9:1236. Originally published 2020 Oct 14. [Version 2] doi: 10.12688/f1000research.26879.2 (PMC7970436; doi:10.12688/f1000research.26879.2)
Supplement: Peer review history

# Peer review: Reviewer response for version 2

**DOI:** 10.5256/f1000research.54199.r80498

**Published:** 2021-03-17

**Authors:** Andrew W. Eller, Saloni Kapoor

**Affiliations:** 1 Retina Service, Department of Ophthalmology, University of Pittsburgh Medical Center (UPMC), Pittsburgh, PA, USA; 2 University of Pittsburgh Medical Center, Pittsburgh, USA

The authors have satisfactorily addressed the comments we provided and made all the changes we suggested. They have not provided a regression analysis as they would like to put this forward in another manuscript which we look forward to reading.

Is the work clearly and accurately presented and does it cite the current literature?

Yes

If applicable, is the statistical analysis and its interpretation appropriate?

Yes

Are all the source data underlying the results available to ensure full reproducibility?

Yes

Is the study design appropriate and is the work technically sound?

Yes

Are the conclusions drawn adequately supported by the results?

Yes

Are sufficient details of methods and analysis provided to allow replication by others?

Yes

Reviewer Expertise:

NA

We confirm that we have read this submission and believe that we have an appropriate level of expertise to confirm that it is of an acceptable scientific standard.

# Peer review: Reviewer response for version 1

**DOI:** 10.5256/f1000research.29684.r75197

**Published:** 2020-12-01

**Authors:** Stefan Dithmar

**Affiliations:** 1 Department of Ophthalmology, HELIOS Dr. Horst Schmidt Kliniken, Wiesbaden, Germany

The authors present a very interesting observational study on the therapy of neovascularization glaucoma. The treatment of this clinical entity is difficult and often unsatisfactory. The authors report on a convincing surgical approach and present retrospective results in a relatively large patient group. The presentation of the results is good and the manuscript is well written.

 Taking into account the reviewer report by Andrew W. Eller, I have no further comments to make.

Is the work clearly and accurately presented and does it cite the current literature?

Yes

If applicable, is the statistical analysis and its interpretation appropriate?

Yes

Are all the source data underlying the results available to ensure full reproducibility?

Yes

Is the study design appropriate and is the work technically sound?

Yes

Are the conclusions drawn adequately supported by the results?

Yes

Are sufficient details of methods and analysis provided to allow replication by others?

Yes

Reviewer Expertise:

Retina research

I confirm that I have read this submission and believe that I have an appropriate level of expertise to confirm that it is of an acceptable scientific standard.

# Peer review: Reviewer response for version 1

**DOI:** 10.5256/f1000research.29684.r73906

**Published:** 2020-11-20

**Authors:** Andrew W. Eller, Saloni Kapoor

**Affiliations:** 1 Retina Service, Department of Ophthalmology, University of Pittsburgh Medical Center (UPMC), Pittsburgh, PA, USA; 2 University of Pittsburgh Medical Center, Pittsburgh, USA

In this study Dr Strzalkowski *et al. *provide a retrospective observational analysis of a single center’s experience with aggressive upfront treatment of neovascular glaucoma (NVG) with combined vitrectomy, near confluent pan retinal endolaser, bevacizumab and transscleral cyclophotocoagulation.

 The authors hypothesize that upfront combined treatment would improve outcomes in terms of IOP, antiglaucoma medications, pain, necessity of further surgeries and help reduce follow-up visits. They report baseline characteristics and one-year outcomes for 77 patients with neovascular glaucoma that underwent the combined procedure. We congratulate them on implementing a success strategy in treating these complex cases. Often patients with NVG have disastrous outcomes with poorly controlled IOP and significant loss of vision. In this series, a number of patients were able to maintain reasonable vision. It can often be difficult to “titrate” treatment of IOP, often resulting in hypotony. There were three patients with phthisis bulbi and another six with hypotony for a total of nine or 11.7%. Three patients developed retinal detachment. Perhaps these were the same three patients who developed phthisis bulbi. 

 The major drawback of this study is the lack of alternate treatments for randomization. This limits our ability to draw any conclusions about optimal management strategies and only hypotheses can be generated. This is appropriately acknowledged by the authors who are careful not to overstate their conclusions.

 The authors seemed to have corelated surgical success with the stage of iris neovascularization. In other words, there were more surgical interventions with stage 4, then stages 2 or 3. It is the experience of this reviewer that one can corelate treatment success of NVG with presenting IOP and the immediate response of IOP to medical therapy. In clinic, if the initial IOP is less than 40 mmHg and medical treatment can reduce the IOP into the low 20’s, these patients can often be treated with an initial injection of an anti-VEGF medication, followed by PRP laser. In the long term, any residual glaucoma can usually be treated with medical therapy alone. Those patients presenting with an IOP greater than 40 mmHg, and not responding to medical therapy will require more aggressive treatment. If the visual potential is reasonable, this treatment may include anti-VEGF, PRP laser (with vitrectomy if visualization of the retina is poor), and glaucoma surgery. If the visual potential is very poor, then not invasive glaucoma treatment such as transscleral CPC) is advised. Using their data, are the authors able to confirm our observations regarding treatment of NVG.

 As the authors have stated, there are a number of different underlying causes of neovascular glaucoma and they have been combined in this series. This is a fairly large series and we question whether the severity of the NVG and response to treatment may have differed according to the underlying pathology. For example, the iris neovascularization and glaucoma in central retinal vein occlusions (CRVO) tends to progress more rapidly then seen with diabetic retinopathy. Once the retinal ischemia has been addressed in CRVO, it seems these eyes are more likely to develop hypotony. What was the experience of the authors in this study?

 The authors cite two studies from the mid-1980’s when the use of silicone oil was very much in its early stages of clinical usage. At that time, it was felt that silicone oil reduced or perhaps prevented the flow of VEGF into the anterior chamber and therefore decreased the risk of iris neovascularization and glaucoma. We believe this theory has been discounted as we know that there is a flow of aqueous humor from the posterior segment into the anterior chamber. Retinal detachment alone increases VEGF production and it occurs in cases of proliferative diabetic retinopathy there can be an even more profound angiogenic effect. An alternative explanation for this observation is that silicone oil improves the outcome in these eyes by simply improving the chances for retinal re-attachment.

 In the Introduction, the authors hypothesize that the combined approach would help decrease outpatient visits. This is again emphasized in the Discussion where authors report that patients with neovascular glaucoma have significant comorbidities and lower life expectancy and would therefore benefit from decreased outpatient visits. The counter-argument to be made is that combined aggressive surgical intervention would increase risk of complications and require closer monitoring. No data is provided on the number of follow up visits. Consider adding to results if available. The authors could consider performing a regression analysis to determine which baseline characteristics predicted success with this combined technique. This analysis will help identify patients in whom this technique would be most helpful. It could also show when this technique may be more aggressive than necessary in some cases, and could be more aggressive in others when the addition of a glaucoma drainage device may be beneficial.

**Specific comments for revision: **

1. In the Introduction , second paragraph, suggest following change. “In 1994, Miller *et al*. showed that laser occlusion of all branch retinal veins in a primate could lead to CRVO and subsequent retinal ischemia.”
2. In the Results, consider rephrasing “arterial hypertension” to hypertension as this is the currently accepted terminology.
3. In Table 1 – consider rephrasing “R stadium” to read “NVI Stage” or Weiss and Gold Rubeosis grading to ensure clarity.
4. In the Discussion, it would be beneficial to see outcomes with staged surgical approach (Incisional surgical management with trabeculectomy/tubes, followed by cryotherapy if poor response) from existing literature to enable some comparison with the combined approach reported in this manuscript.
5. In the Discussion, the authors state “Patients with stage 4 anterior chamber neovascularization needed significantly more major interventions than stage 3 or stage 2, but there was a difference between stage 2 and stage 3”. The data reports NO significant difference between stage 2 and 3. Please clarify.

Overall, a well-organized manuscript with a practical approach to the treatment of neovascular glaucoma with novel data.

Is the work clearly and accurately presented and does it cite the current literature?

Yes

If applicable, is the statistical analysis and its interpretation appropriate?

Yes

Are all the source data underlying the results available to ensure full reproducibility?

Yes

Is the study design appropriate and is the work technically sound?

Yes

Are the conclusions drawn adequately supported by the results?

Yes

Are sufficient details of methods and analysis provided to allow replication by others?

Yes

Reviewer Expertise:

Ophthalmology, Vitreo-Retinal Diseases and Surgery

We confirm that we have read this submission and believe that we have an appropriate level of expertise to confirm that it is of an acceptable scientific standard, however we have significant reservations, as outlined above.

# Response

**Published:** 2021-01-23

**Authors:** Piotr Strzalkowski

**Affiliations:** University Hospital Würzburg, Germany

**Dear Reviewers,**

 We are grateful for the opportunity to improve our manuscript. We appreciate the helpful comments and have provided our replies below. We trust that we were able to address the concerns that were raised.

 Thank you,

 Piotr Strzalkowski

**Reviewers' comments:**

**Reviewer #1 (“R1” in the following): **There were three patients with phthisis bulbi and another six with hypotony for a total of nine or 11.7%. Three patients developed retinal detachment. Perhaps these were the same three patients who developed phthisis bulbi.

**Authors: **That is exactly correct. We appreciate the thoughtful analysis. All three patients with retinal detachment also developed phthisis bulbi. We added this important information to our manuscript.

**R1, 1:** As the authors have stated, there are a number of different underlying causes of neovascular glaucoma and they have been combined in this series. This is a fairly large series and we question whether the severity of the NVG and response to treatment may have differed according to the underlying pathology. For example, the iris neovascularization and glaucoma in central retinal vein occlusions (CRVO) tends to progress more rapidly than seen with diabetic retinopathy. Once the retinal ischemia has been addressed in CRVO, it seems these eyes are more likely to develop hypotony. What was the experience of the authors in this study?

**Authors:** Thank you for sharing your clinical experience. Based on our data we could not find a statistical significant difference in postoperative IOP or hypotony between CRVO and PDR patients.

**R1, 2: **No data is provided on the number of follow up visits. Consider adding to results if available.

**Authors: **Information about the number of follow up visits is provided in section “study design”:  All patients were treated at the University Eye Hospital in Würzburg, Germany. The combined intervention was part of routine patient care at our clinic and all patients with decompensated neovascular glaucoma and preserved visual function received this intervention. BCVA, intraocular pressure (IOP, mmHg), the number of glaucoma medication, visual analog pain scale (VAPS, 0-10) was recorded at baseline and at follow-up visits at one, three, six, and 12 months as part of routine care.

**R1, 3:** The authors could consider performing a regression analysis to determine which baseline characteristics predicted success with this combined technique. This analysis will help identify patients in whom this technique would be most helpful.

**Authors: **Regression analysis was not included in this study on purpose because this analysis is part of our ongoing study.

**R1, Specific Comments:**

- In the Introduction, second paragraph, suggest following change. “In 1994, Miller et al. showed that laser occlusion of all branch retinal veins in a primate could lead to CRVO and subsequent retinal ischemia.”
- In the Results, consider rephrasing “arterial hypertension” to hypertension as this is the currently accepted terminology.
- In Table 1 – consider rephrasing “R stadium” to read “NVI Stage” or Weiss and Gold Rubeosis grading to ensure clarity.
- In the Discussion, it would be beneficial to see outcomes with staged surgical approach (Incisional surgical management with trabeculectomy/tubes, followed by cryotherapy if poor response) from existing literature to enable some comparison with the combined approach reported in this manuscript.
- In the Discussion, the authors state “Patients with stage 4 anterior chamber neovascularization needed significantly more major interventions than stage 3 or stage 2, but there was a difference between stage 2 and stage 3”. The data reports NO significant difference between stage 2 and 3. Please clarify.

**Authors: **We have changed the manuscript to include these suggestions.
